# *Pterocarpus marsupium* Roxb. heartwood extract synthesized chitosan nanoparticles and its biomedical applications

**DOI:** 10.1186/s43141-020-00033-x

**Published:** 2020-07-06

**Authors:** Anupama Ammulu Manne, Vinay Viswanath K., Ajay Kumar G, Ushakiranmayi Mangamuri, Sudhakar Podha

**Affiliations:** 1 Department of Biotechnology, Acharya Nagarjuna University, Nagarjuna nagar, Guntur, Andhra Pradesh 522510 India; 2 Department of Botany and Microbiology, Acharya Nagarjuna University, Nagarjuna nagar, Guntur, Andhra Pradesh 522510 India

**Keywords:** Chitosan nanoparticles, *Pterocarpus marsupium*, Alpha-amylase inhibitory activity, Anti-inflammatory activity

## Abstract

**Background:**

The point of the present investigation was to blend effective chitosan nanoparticles (CNPs) loaded with *Pterocarpus marsupium* (PM) heartwood extract and evaluate its biomedical applications. Various plant extract concentrations (PM-CNPs-1, PM-CNPs-2, PM-CNPs-3) are used to synthesize chitosan nanoparticles and optimized to acquire a stable nanoparticle formulation. The entrapment efficiency and in vitro release studies of the plant extract encapsulated in CNPs are estimated. The PM-loaded CNPs were characterized by X-ray diffraction, dynamic light scattering (DLS), Fourier transform infrared spectroscopy (FT-IR), scanning electron microscopy (SEM), and transmission electron microscopy (TEM). The synthesized chitosan nanoparticles were evaluated for their alpha-amylase inhibitory activity and inhibition of albumin denaturation activity.

**Results:**

The XRD pattern of PM-CNPs shows less number of peaks at low intensity due to the interaction of chitosan with sodium tripolyphosphate. The FT-IR spectrum with peaks at 1639.55 and 1149.02 cm^−1^ confirms the formation of chitosan nanoparticles. The size of the nanoparticles ranges between 100 and 110 nm with spherical shape illustrated by SEM and TEM analysis. The nanoparticle formulation with 10% plant extract concentration (PM-CNPs-2) showed optimum particle size, higher stability, enhanced entrapment efficiency, and sustained drug release characteristics. Synthesized chitosan nanoparticles have shown a significant increase in alpha-amylase inhibition and appreciable anti-inflammatory activity as measured by inhibition of protein denaturation.

**Conclusions:**

The investigation reports the eco-friendly, cost-effective method for synthesizing chitosan nanoparticles loaded with *Pterocarpus marsupium* Rox.b heartwood extract.

## Background

Nanotechnology is the new science dealing with nanometer (nm) size, and nanoparticles (NPs) are one of the nanotechnology building blocks [1–3]. Nanotechnology combined with polymers has recently captivated immense interest in many fields including the pharmaceutical industry and medical field. NPs are the nanometer-ranging stable colloidal particles, i.e., from 10 to 1000 nm [4]. They show unusual physical and chemical properties, owing to their smaller size and larger surface area. Nanoparticles can be made from naturally occurring polymers like protein, polysaccharide or synthetic polymer like polystyrene. Nanoparticles made from natural polymers, by comparison, give both mild and easy preparation procedures without using organic solvent and higher shear force. Due to their intrinsic biological properties, chitosan nanoparticles have gained significant interest in the current scenario over the past years.

Chitosan nanoparticles (CNPs) are used in a range of various products and applications, ranging from medicinal, drug delivery, tissue engineering, and food processing to bio-sensing, immobilization of enzymes, fuel cell development, and wastewater treatment [5]. Chitosan [poly(b-1/4)-2-amino-2-deoxy-D-glucopyranose] is a biopolymer which is a chitin derivative, extracted from the shellfish exoskeleton [6–8]. Chitosan has properties forming a film, fiber, and micro/nanoparticle, for its abundance, low cost of manufacturing, biodegradability, biocompatibility, reusable, and non-toxic nature. Because of their capacity to provide safety and stability, chitosan nanoparticles were used in the delivery of active components. Recently, extensive research has been carried out on chitosan nanoparticles to target biomolecules including anticancer chemotherapy [9], antibiotics [10], vaccines [11], peptides, genes [12], etc.

Polymers like chitosan nanoparticles are ideal shielding agents and can be used to deliver active ingredients. To date, CNPs ranging from few to several hundred nanometers have been manufactured using chemosynthetic techniques such as microemulsion, oil-water emulsion, spray-drying, precipitation, complex formation of polyelectrolytes, and methods of desolvation [13]. Nevertheless, due to the high solution viscosity and coiled nature of high molecular weight chitosan (HMWC), these synthetic techniques result in the creation of microparticles of more than 1000 nm. The entry of drug-charged particles into cells with pore sizes below 1000 nm in vivo is severely limited. It was calculated that the efficiency of cellular absorption of polymeric particles with a diameter of less than 1000 nm was 2.5–250 times greater than particles exceeding the nanometer scale [14]. Chemosynthetic techniques often rely on stabilizing agents such as polyethylene glycol (PEG), polyvinyl alcohol, and succinate D-alpha-tocopheryl poly (ethylene glycol) [15]. Such additional surface stabilizers significantly improve the half-life of nanoparticles by enhancing the longevity of NPs in the blood, which can induce nanotoxicity [16].

Eventually, regulating the size and shape of nanoparticles by enhancing current physicochemical techniques was seen as a priority for material scientists to reduce, size-oriented limitations of polymeric nanoparticles. In this context, green nanotechnology, a well-established interdisciplinary nanoscience branch, has shown huge potential for creating nanostructures that are strictly within the range of a few nanometers, using biological systems like plants, fungi, and bacteria [17, 18]. Cross-linking chitosan (CS) with sodium tripolyphosphate (TPP) is a moderate, efficient approach for achieving CNPs. The nanoparticles are formed by the ionic gelation method, where electrostatic interaction between positively charged CS and negatively charged TPP molecules are cross-linked to form a chitosan nanoparticle.

Chitosan nanoparticles were loaded with various plants such as *Arrabidaea chica* [19], *Mentha longifolia* [20], *Leucas aspera* [21], and others. The physicochemical properties of chitosan nanoparticles such as size, surface area, cationic composition, effective functional groups, high permeability towards biological membranes, non-toxicity to individuals, cost efficiency, higher encapsulation performance and/or by mixing with other compounds and wide-ranging antifungal and antimicrobial activities have resulted in increased use in biomedical applications [22–25]. Thus, due to the extensive applications, chitosan polymer was chosen for the present research.

*Pterocarpus marsupium* plant belongs to the Fabaceae family and because of its diverse biological activities since ancient times it has been used in India and its neighboring countries. All parts of the plant are used as primitive home remedies against various human diseases. This was commonly used in homeopathic medicine, Unani systems, and ayurvedic [26, 27]. It is also commonly referred to as Malabar kino or Indian kino tree or Vijayasar, it is a medium to large deciduous tree which can grow up to 30 m (98 ft.) tall [28, 29]. This is native to India, Nepal, and Srilanka. Heartwood (stem bark) is golden-yellow in color, possessing antidiabetic, astringent, anti-inflammatory, hypertriglyceridemic, cardiotonic, wound healing, hepatoprotective function, and as a potent COX-2 inhibitor [30–33]. The plant *P. marsupium* was recognized as a very rich source of flavonoids and polyphenolic substances by earlier researchers [34]. All of *P. marsupium* functional phytoconstituents were thermostable. An active compound, epicatechin, was detected from the PM bark ethanol extract and the presence of three phenolic compounds pterostilbene, pterosupin, and marsupin as antidiabetic agents in ethylacetate-soluble portions of aqueous extract of PM heartwood was reported [35]. Traditionally different plants have been used as anti-inflammatory agents. In Indian medicine *P. marsupium* was used from ancient times to treat burns, psoriasis, wounds, odontalgia, etc. [36]. The most significant biologically active ingredients in stem bark were terpenoids, saponins, tannins, alkaloids, flavonoids, and phenolic compounds. Due to various pharmacological activities, the plant *Pterocarpus marsupium* was taken in the present research.

The current research has been conducted to formulate and establish strategies to increase the therapeutic potential of standardized *Pterocarpus marsupium* heartwood extract using CNPs as carriers. The parameters have been modified to produce the optimal output of NPs. Introducing nanotechnology to plant extracts has provided a beneficial strategy for herbal medicinal products, taking into account the several features to be offered by nanostructured systems includes increasing solubility, pharmacological activity, bioavailability, safety from toxicity, controlled delivery, and safety from physical and chemical degradation [37, 38]. At present, processes in nanotechnology involving medicinal plants have produced several novel delivery methods, including polymeric nanoparticles. Those made from biocompatible and biodegradable polymers such as chitosan (CS) provide an alternative for sustained drug delivery [39]. These prepared green synthesized chitosan nanoparticles were characterized for size and morphology, drug entrapment efficiency, and drug release studies. These are evaluated for their anti-diabetic and anti-inflammatory activities.

## Methods

### Preparation of aqueous extract

The plant *Pterocarpus marsupium* Roxb. heartwood was collected from in and around Tirupati area and authenticated by Dr. K. Madhava Chetty, Assistant Professor, Department of Botany, Sri Venkateswara University, Tirupati (voucher no. 1587 has been deposited in a herbarium), dried PM heartwood was ground to fine powder, and 20 g of which was boiled in 100 ml of distilled water for 20 min. The obtained aqueous extract was then cooled and filtered using Whatman No. 1 filter paper, and kept in the refrigerator for further use.

### Synthesis of nanoparticles

Chitosan nanoparticles were prepared by ionic gelation method [40]. Chitosan solution was prepared by adding 40 mg of chitosan (deacetylation degree ≥ 85%, M) to 1% (v/v) acetic acid slowly in smaller amounts for higher solubility in acidic medium. By using a digital homogenizer the solution was continuously stirred at 7000 rpm to obtain a homogeneous solution. The CS solution should maintain a pH of 5. Various concentrations of 5%, 10%, and 15% (PM-CNPs-1, PM-CNPs-2, PM-CNPs-3) of plant aqueous extract was added concerning to the chitosan concentration to the homogenous solution to obtain optimized nanoparticles. For cross-linking of chitosan as nanoparticles, an ionic crosslinking agent, sodium tripolyphosphate (TPP) was used, 8 ml of 0.1% TPP solution prepared in distilled water was introduced in dropwise to chitosan solution while homogenizing at a constant speed of 7000 rpm at room temperature for 2 h. Finally, the PM-CNPs so formed were separated from the solution by centrifugation at 15000 rpm for 30 min and freeze-dried to obtain nanoparticles in powder form.

### Characterization of nanoparticles

#### X-ray diffraction

X-ray diffraction (XRD) technique was used to classify the prepared PM-CNPs by using an X-ray diffractometer (Miniflex 600 Powder XRD, Osmania University). The calculation was carried out at a scanning speed of 50 S^−1^ and within the scanning range of 10–80.

### Fourier transform infrared spectroscopy

Fourier transform infrared spectroscopy was executed to analyze the formation of chitosan nanoparticles. The spectra of the considerable number of test samples (PM, CNPs, PM-CNPs) were recorded spectrometrically (Shimadzu FT-IR spectrophotometer) by KBr pellet development at room temperature. The range was kept in the range of 4000 and 400 cm^−1^ at the resolution of 4 cm^−1^.

### Zeta potential, particle size, and PDI

The particle size and zeta potential of PM-CNPs were analyzed using a laser light scattering-based particle size analyzer (Zetasizer NS 3000, Malvern Instruments). The analysis was performed in triplicate manner, and average values with standard deviation were recorded.

### Shape and surface morphology

The morphology of the arranged PM-CNPs was analyzed by scanning electron microscopy (SEM), using Zeiss SEM machine and transmission electron microscopy (TEM) using FEI-Tecnai G2 20 Twin, VIT University. The test sample (CNPs and PM-CNPs) for SEM investigation was set up by placing the filtered lyophilized nanoparticles onto the network, permitted to dry under a mercury light for SEM analysis after 10 min of drying. Test for TEM investigation was set up by placing a little drop of nanoparticle suspension on a carbon-covered copper network and enabling water to dissipate in a vacuum dryer. The grid containing PM-CNPs was scanned for TEM images.

### Entrapment efficiency

The measure of PM encapsulated in the nanoparticles was controlled by UV spectrophotometry (Shimadzu UV-160 spectrophotometer, Kyoto, Japan). Each sample containing various concentrations of PM extract was centrifuged at 15000 rpm for 30 min and the supernatants are analyzed for absorbances at *λ*_max_ of 279 nm using a UV spectrophotometer [41]. Values of absorbances acquired were quantified in mg of the plant extract present in the supernatant. This percentage of plant extract entrapped was calculated with the initial amount of the PM in the nanoparticles. Encapsulation efficiency of plant extract was determined by the following equation

$$ \mathrm{EE}\ \left(\%\right)=\frac{\mathrm{mass}\ \mathrm{of}\ \mathrm{initially}\ \mathrm{added}\ \mathrm{drug}-\mathrm{mass}\ \mathrm{of}\ \mathrm{free}\ \mathrm{drug}}{\mathrm{mass}\ \mathrm{of}\ \mathrm{initially}\ \mathrm{added}\ \mathrm{drug}}\times 100 $$

The influence of plant extract concentration on EE was performed. The optimized nanoparticle formulation was taken for further in vitro drug release studies.

### In vitro drug release studies

Studies of in vitro drug discharge were conducted using the technique of tube dialysis for the prepared formulations [42]. Two milligrams of freeze-dried PM-CNPs-2 were spread into the individual dialysis tube (MW 12,000 Da, Himedia) in 10 ml of phosphate buffer saline. The sealed dialysis bag was immersed in a 30 ml PBS (Ph 7.4) buffer at 100 rpm at room temperature with constant stirring. A 2 ml aliquot was collected at regular time intervals and a similar quantity of PBS was added to the release medium. The aliquots were evaluated as a function of time using a UV spectrophotometer for the drug release concentration. The drug release concentration has been determined by using the following equation

$$ \mathrm{Drug}\ \mathrm{release}\ \left(\%\right)=\frac{\mathrm{mass}\ \mathrm{of}\ \mathrm{encapsulated}\ \mathrm{drug}-\mathrm{mass}\ \mathrm{of}\ \mathrm{release}\ \mathrm{drug}}{\mathrm{mass}\ \mathrm{of}\ \mathrm{encapsulated}\ \mathrm{drug}}\times 100 $$

### Assay of α-amylase inhibition

Alpha-amylase inhibitory activity of PM incorporated chitosan nanoparticles was examined by employing the method determined by Poongunran [43]. Quickly, 100 μl of the integrated chitosan nanoparticles (PM-CNPs-2) were allowed to react with 200 μl of α-amylase and 100 μl of 2 mM phosphate buffer (pH 6.9). To which 100 ml of 1% starch solution was added and incubated for 20 min. The equivalent was employed to act as control where 200 μl of the enzyme was substituted with buffer. After incubating for 10 min, 500 μl of the DNS reagent was added to both control and test. They were subjected to boiling in a water bath for 5 min. The optical density was recorded at 540 nm utilizing a spectrophotometer. The percentage inhibition of α-amylase enzyme was determined by using the following formula

$$ \mathrm{Inhibition}\ \left(\%\right)=100\times \left[\mathrm{Control}-\mathrm{Test}/\mathrm{Control}\right] $$

### Inhibition of protein denaturation

The synthesized compounds are tested for anti-inflammatory activity using the albumin denaturation inhibition method [44]. The standard drug (diclofenac sodium) and synthesized nanoparticles (PM-CNPs-2) were dissolved in dimethylformamide (DMF) and diluted with phosphate buffer (0.2 M, pH 7.4). The final concentration of DMF was < 2.5% in all alternatives. Test solution (1 ml) containing different nanoparticle concentrations was mixed in phosphate buffer with 1 ml of 1 mM albumin solution and incubated in the BOD incubator at 27 ^0^C ± 1 ^0^C for 15 min. Denaturation was induced by 10 min in a water bath keeping the reaction mixture at 60 ^0^C ± 10 ^0^C. The turbidity at 660 nm (UV-visible spectrophotometer, Shimadzu) was evaluated after cooling. The percentage of albumin denaturation inhibition was calculated from the control, where no drug was added. All the experiments were conducted in triplicate.

### Statistical evaluation

All results are expressed as mean ± SEM (*n* = 6). IC_50_ values were calculated by applying a suitable regression analysis of the mean inhibitory values.

## Results

### X-ray diffraction

**Fig. 1:**
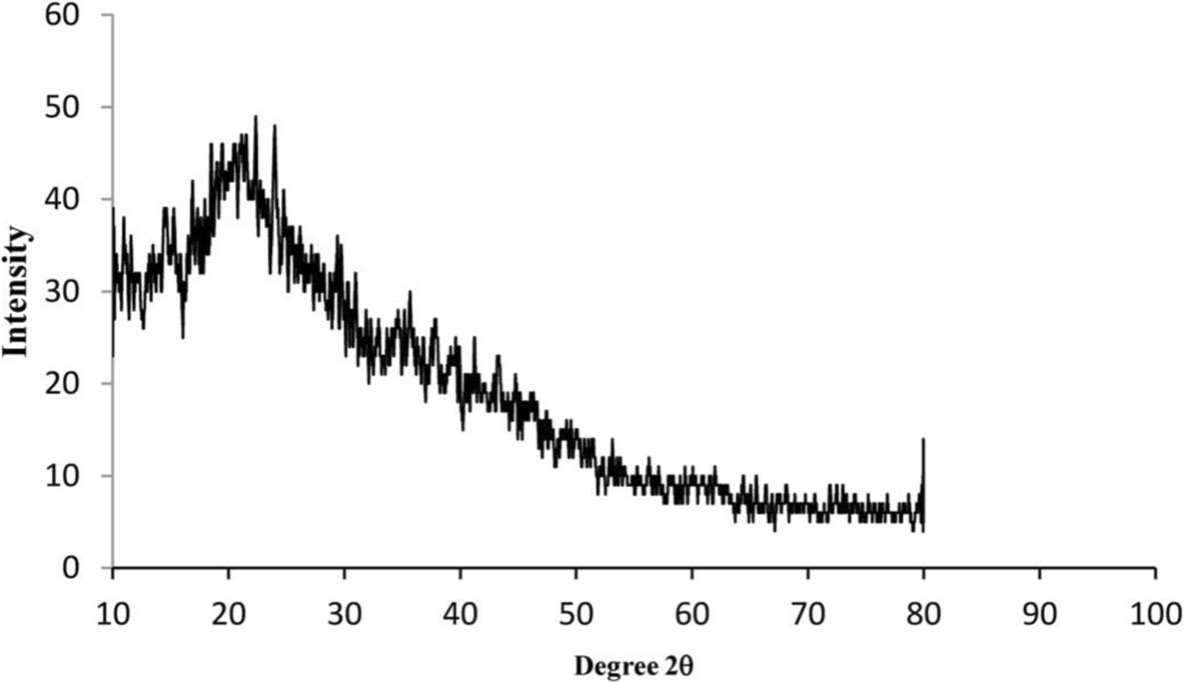
XRD pattern of chitosan nanoparticles synthesized by *Pterocarpus marsupium* Rox.b heartwood extract

Figure 1 represents the pattern of chitosan nanoparticles loaded with PM extract in X-ray powder diffraction. Less noticeable peaks with very low intensity in the diffractogram of PM-CNPs showing a complex network structure of interpenetrating polymer chains of CS cross-linked with one another by TPP counter ions. While this diffractogram with lower peaks showed the formation of chitosan nanoparticles with heavy interaction between the counter-ions of chitosan and TPP.

### Zeta potential, particle size, and PDI

The ZP represents the superficial electrical charge of particles and is determined by the composition of the particles and the dispersion media. This parameter is used as an index of NP stability. The electronic repulsion among particles can significantly affect the stabilization of the suspended particles. Consequently, a higher absolute zeta-potential value suggests a more stable suspension, while a lower value means colloidal instability, which may lead to aggregation of nanoparticles. Nanoparticles with a zeta potential > ± 30 mV are stable in suspension, as the surface charge inhibits particle aggregation [45].

**Fig. 2:**
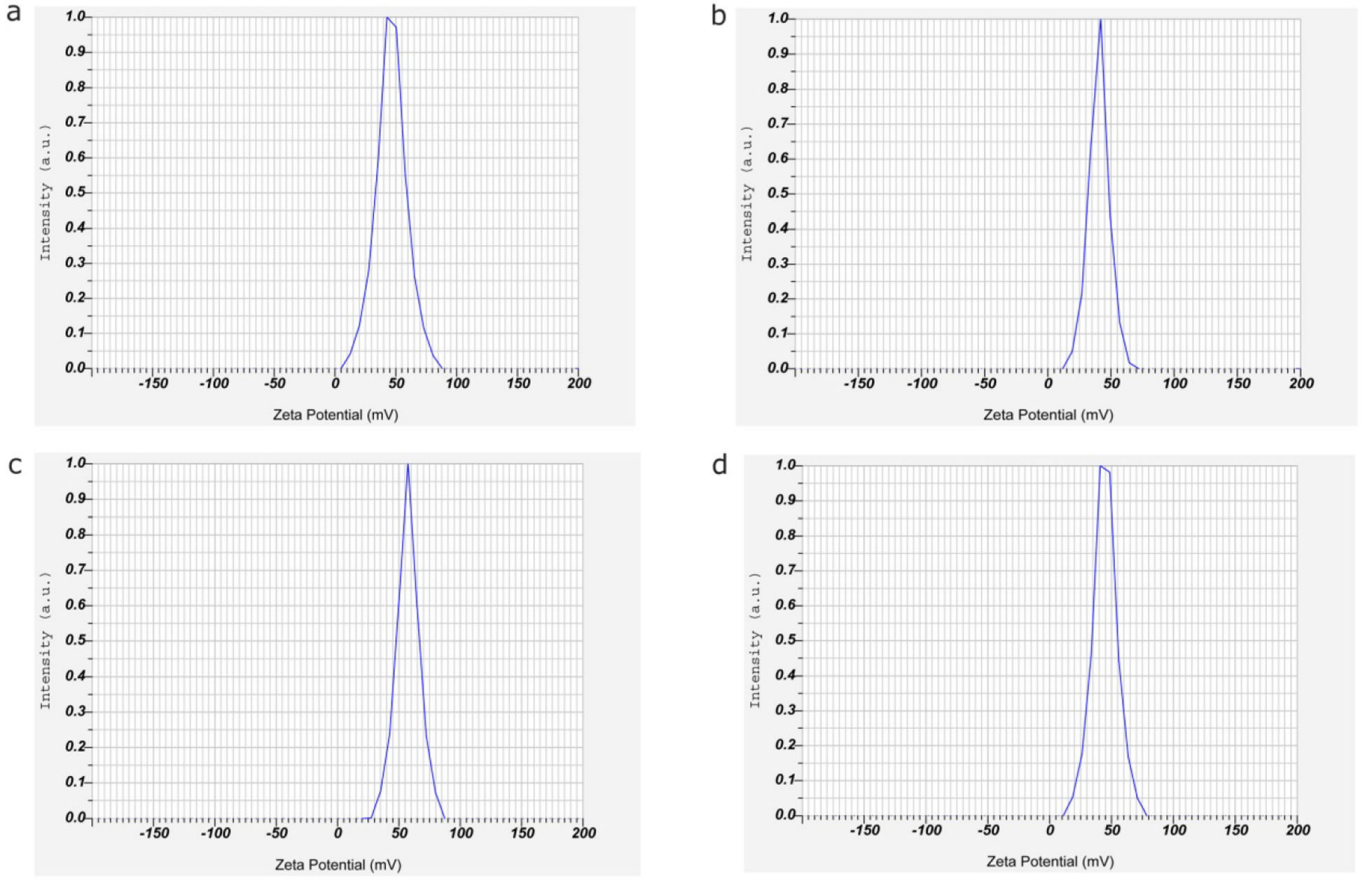
Zeta potential distribution of chitosan nanoparticles (CNPs) loaded with *Pterocarpus marsupium* (PM) stem bark extract concentrations. **a** CNPs. **b** PM-CNPs-1. **c** PM-CNPs-2. **d** PM-CNPs-3

**Fig. 3:**
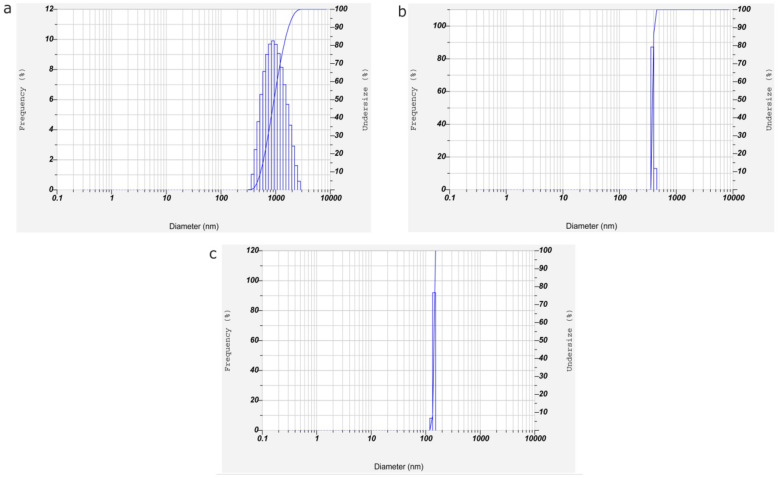
Particle size distribution of chitosan nanoparticles (CNPs) loaded with different *P. marsupium* stem bark extract concentrations. **a** PM-CNPs-1. **b** PM-CNPs-2. **c** PM-CNPs-3

The nanoparticle formulation, PM-CNPs-2 resulted in enhanced effectiveness of drug trapping with optimum mean particle size and PDI for chitosan nanoparticles. Thus PM-CNPs-2 with 57.3 mV and with an optimum particle size of 676± 2.76 nm are stable compared to other plant extract concentration-loaded nanoparticles as shown in Figs. 2 and 3.

### FT-IR spectroscopy analysis of chitosan nanoparticles

**Fig. 4:**
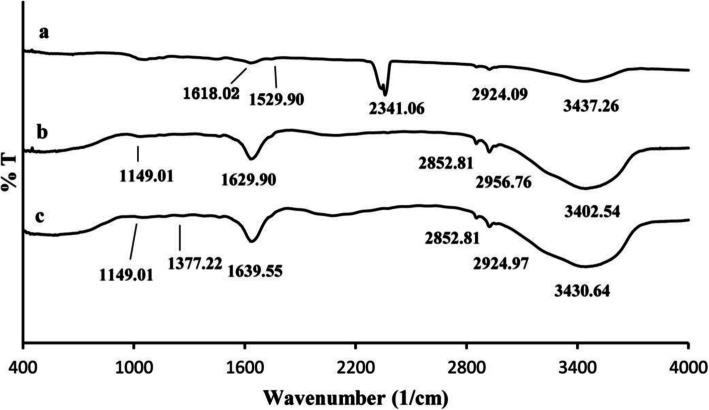
FT-IR spectrums of **a***Pterocarpus marsupium* (PM) aqueous stem bark extract, **b** chitosan nanoparticles (CNPs), and **c***Pterocarpus marsupium-*loaded chitosan nanoparticles (PM-CNPs)

Chitosan nanoparticles were synthesized by cross-linking chitosan and tripolyphosphate (TPP). FT-IR studies of chitosan nanoparticles (CNPs), plant extract (PE), and PM extract-loaded chitosan nanoparticles (PM-CNPs) were performed to characterize the chemical nature of the nanoparticles as shown in the Fig. 4. The present studies showed sharp absorption peaks at 3430.64, 2956.97, 1629.90, 1149.01, and 1035.81 cm^−1^ for chitosan nanoparticles (Fig. 4b) and intense peaks were found at 3402.54, 2924.18, 1639.55, 1464.02, 1377.22, 1149.01, 1047.36 cm^−1^ for PM-CNPs (Fig. 4c). FT-IR study for *Pterocarpus marsupium* stem bark aqueous extract showed sharp peaks at 3437.26, 3429.56, 2924, 2232.70, 2341.06, 1618.02, and 1529.90 cm^−1^ (Fig. 4a). The results confirmed that the biomolecules of PM stem bark extract were loaded in chitosan nanoparticles.

### Shape and surface morphology

**Fig. 5:**
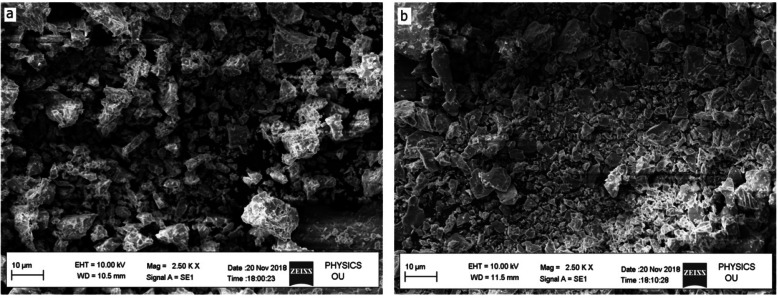
SEM micrographs of **a** chitosan nanoparticles at 10 μm. **b***Pterocarpus marsupium* extract-loaded chitosan nanoparticles at 10 μm scale

**Fig. 6:**
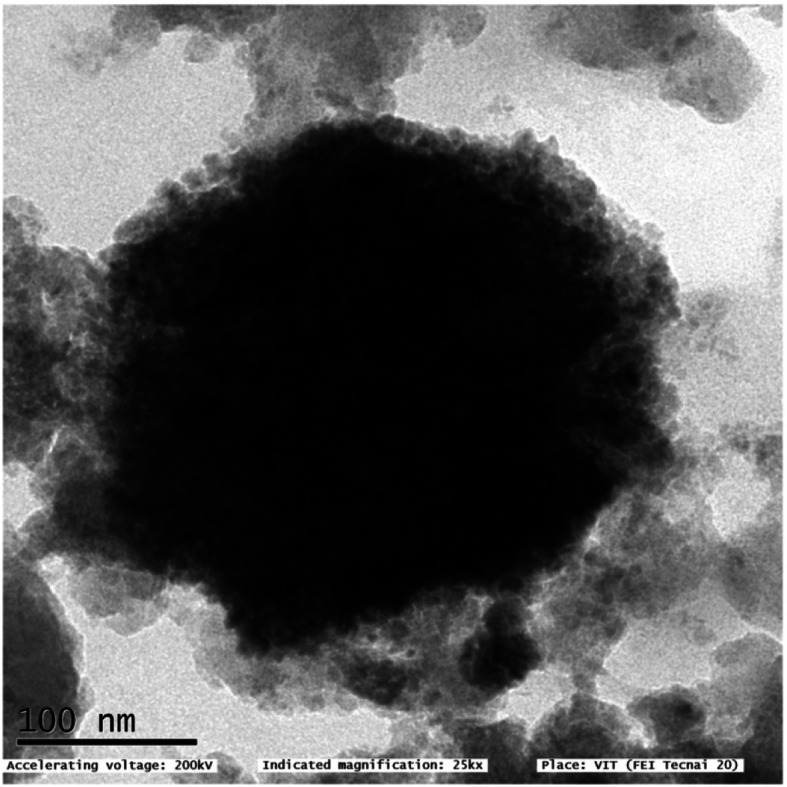
TEM micrograph of *Pterocarpus marsupium-*loaded chitosan nanoparticles at 100 nm magnification

Figure 5 represents the SEM images of *P. marsupium-*loaded and *P. marsupium*-unloaded chitosan nanoparticles at 10 μm scale bar. The SEM pictures confirm the formation of chitosan nanoparticles. The images clearly show the spherical shape of the nanoparticles. The TEM image also portrays the spherical shape of the nanoparticles as shown in Fig. 6.

### Entrapment efficiency

Entrapment efficiency of PM-loaded nanoparticles was estimated and performed in triplicate. The effect of the amount of PM on EE is shown in Table 1. The formulation of nanoparticles showed an improvement in the effectiveness of trapping with a rise in the concentration of drugs. The entrapment efficiencies of PM-CNPs-1, PM-CNPs-2, and PM-CNPs-3 are 68.87 ± 3.1, 82.78± 5.67, and 86.76± 5.43.

### In vitro drug release studies

**Fig. 7:**
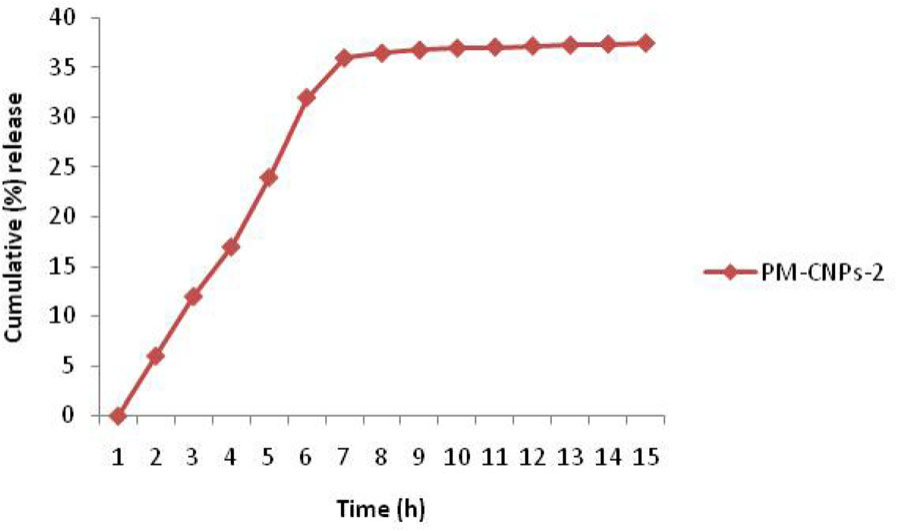
Drug Release profile of *Pterocarpus marsupium* extract-loaded chitosan nanoparticles

The in vitro PM release from PM-CNPs-2 is demonstrated in Fig. 7 from which it was proven that the release of the drug from chitosan nanoparticles was sustained throughout 15 h with a cumulative release of 37.5%.

### Effect on α-amylase inhibition

**Fig. 8:**
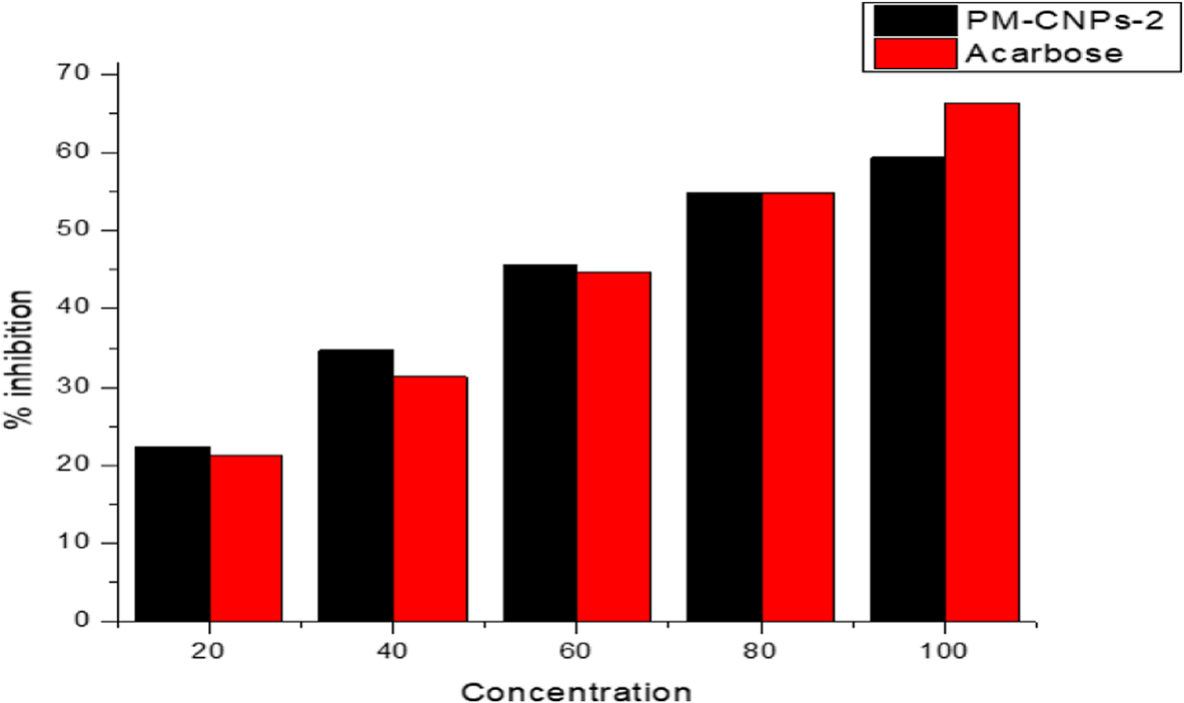
Representing alpha-amylase inhibition evaluated by treating with various concentrations of PM-CNPs-2 and Acarbose

Figure 8 demonstrates the activity of PM-CNPs-2 on inhibiting amylase. The percentage inhibition of α-amylase by the chitosan nanoparticle synthesized with *P. marsupium* was researched within a concentration range of 20–100 μg/ml and 62.46 was found to be the IC_50_ value as shown in Table 2.

### Effect on protein denaturation

**Fig. 9:**
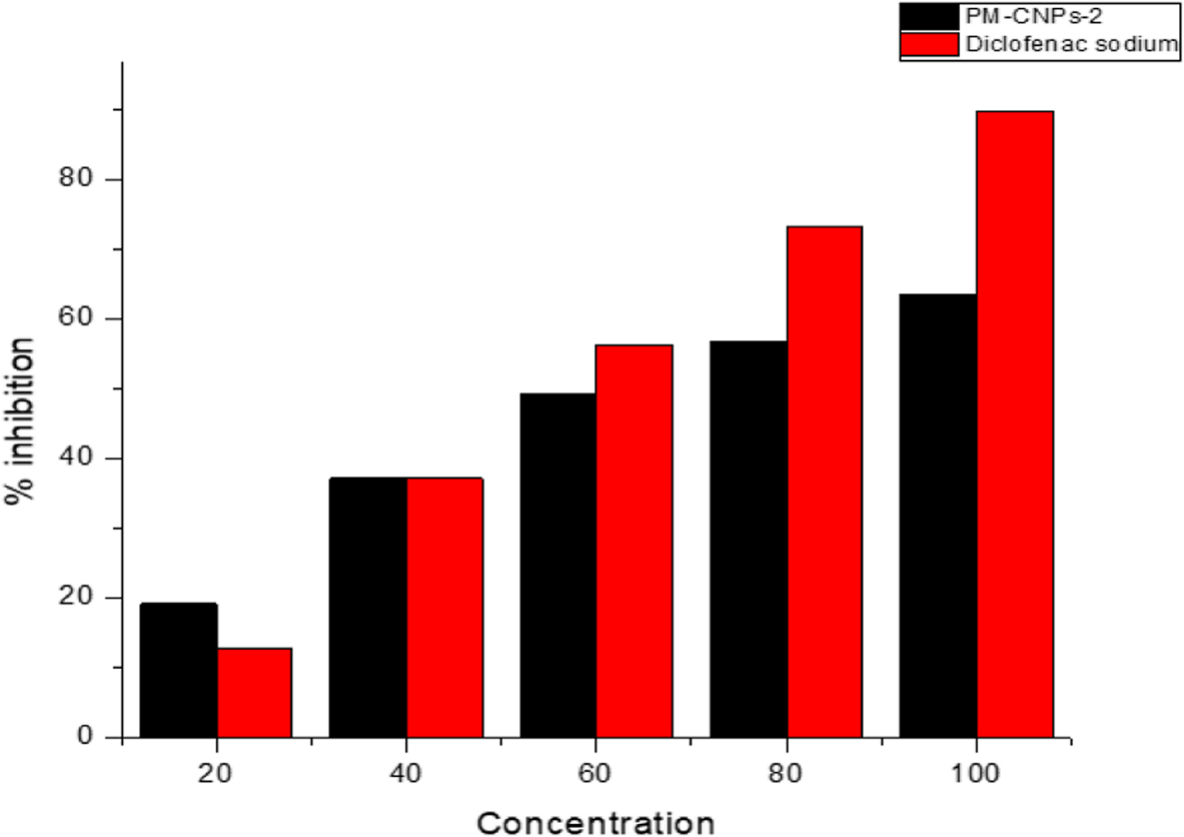
Albumin inhibitory activity evaluated by treating with various concentrations of PM-CNPs-2 and diclofenac sodium

Figure 9 illustrates the in vitro anti-inflammatory activity of PM-CNPs-2 on inhibiting protein denaturation. The minimum inhibition observed at 20 μg/ml concentration by the synthesized nanoparticles is 19.23 ± 1.07%. The IC_50_ was found to be 92.76 as shown in Table 3.

## Discussion

### X-ray diffraction studies

The XRD analysis shows the presence of chitosan nanoparticles with characteristic diffraction peaks (Fig. 1). The XRD pattern of chitosan nanoparticles synthesized from *P. marsupium* Rox.b heartwood extract showed a large diffraction peak at 2*θ* range of 22.56^°^, respectively, which is a standard fingerprint of CNPs. The lower intensity exhibited by PM-CNPs diffraction peaks shows that they are amorphous in nature. In the XRD patterns of PM-loaded chitosan nanoparticles, the absence of any other diffraction peaks, corresponding to impurities is observed, which indicates their purity. The rate of diffraction peaks is reduced in the case of chitosan nanoparticles, as a result of transforming crystallized chitosan into amorphous form after being cross-linked with tripolyphosphate. In our analysis, the diffraction peak of pure chitosan usually observed at 22.04^°^ [46] has shifted slightly to a higher value of 22.56^°^, and this can be due to the cross-linking of chitosan nanoparticles with TPP and the amorphous chitosan structure.

### Zeta potential, particle size, and PDI

Increasing the drug quantity improved the nanoparticle particle size and PDI. Chitosan nanoparticles are synthesized by linking cationic ammonium groups of chitosan and anionic phosphate groups of tripolyphosphate (TPP). The surface charge on the chitosan nanoparticles are indicated by the number of unneutralized ammonium groups in chitosan. Since the number of positive charge groups of chitosan is more than the number of negative charges of TPP, the net charge on the chitosan nanoparticles is positive.

The average size, zeta potential, and polydispersity index of the chitosan nanoparticle formulations were evaluated by zeta sizer analysis as shown in Table 4. The average sizes of CNPs, PM-CNPs-1, PM-CNPs-2, and PM-CNPs-3 were 238 ± 145, 445 ± 2.43, 676 ± 2.76, and 547 ± 286 nm. respectively as shown in Fig. 3. Due to the interaction between the polymer and plant extract structure, the particle size increases with a rise in plant extract concentration. The value of the polydispersity index (PDI), varying from 0 to 1, determines the particle homogeneity. All formulations polydispersity index values ranged from 0.276 to 0.465. As observed in Fig. 2, the zeta potential values indicate that the surface of the chitosan nanoparticle without plant extract had a positive charge of 36.2 mV, and the plant extract-loaded chitosan nanoparticles at various plant extract concentrations (PM-CNPs-1, PM-CNPs-2, PM-CNPs-3) exhibited zeta potentials of 40.4, 57.3, 44.6 mV, respectively. The PM-CNPs had higher positive value ranges from 40.0 to 57.3 mV compared to CNPs (36.2mV), this is because of the rise in groups of positive charges on the surface of plant extract synthesized chitosan nanoparticles. The result showed that PM-CNPs-2 had a Zeta potential of 57.3 mV. The higher ZP indicates CNPs are relatively stable. The long amino groups seem likely to inhibit anion adsorption and hold the value of the electrical double layer thickness high, thus preventing aggregations.

**Table 1:** Entrapment efficiencies of different concentrations of *Pterocarpus marsupium* heartwood extract

Plant extract	Entrapment
concentration (%)	efficiency
2.5	48.78± 2.71
5	68.87± 3.1
10	82.78± 5.67
15	86.76± 5.43
20	86.56± 5.32

### FT-IR spectroscopy analysis of chitosan nanoparticles

Chitosan nanoparticles were synthesized by cross-linking chitosan and tripolyphosphate (TPP). FT-IR studies of chitosan nanoparticles, plant extract, plant extract-loaded chitosan nanoparticles were performed to characterize the chemical nature of the nanoparticles. The FT-IR analysis was carried out to determine the biomolecules specifically bound on the chitosan nanoparticles that were involved in the stabilization. FT-IR spectra of chitosan nanoparticles and PM-CNPs were prepared are represented in Fig. 4b, c showing the peaks of NH_2_ and OH group stretching vibrations in chitosan at 3430 and 3402 cm^−1^. The amount of water present in the chitosan nanoparticles provides an indication of its hydrophilic existence, which is consistent with the –OH and –NH_2_ bonding observed in the FT-IR spectrum of the chitosan nanoparticles between 3430.64 and 3402 cm^−1^ [47, 48]. In PM-CNPs, a shift was observed from 3430 to 3402 cm^−1^ and this peak at 3402 cm^−1^ is less wide, indicating reduced hydrogen bonding. The decreased binding of hydrogen was due to a more open framework arising from interconnection with biomolecules of *Pterocarpus marsupium* extract as well as TPP. Table 5 shows all the peaks of PM-CNPs and its functional groups.

**Table 2:** Alpha-Amylase inhibitory activity by PM-CNPs-2

Treatment	Concentration (μg/ml)	Percentage of inhibition	IC_50_
Chitosan nanoparticles	20	22.45 ± 1.21	62.46
Loaded *Pterocarpus*	40	34.67 ± 1.33	
*Marsupium*	60	45.58 ± 1.53	
	80	54.76 ± 1.65	
	100	59.34 ± 1.63	
Acarbose	20	21.23 ± 1.04	236.78
	40	31.34 ± 1.68	
	60	44.65 ± 1.54	
	80	54.87 ± 1.67	
	100	66.32 ± 2.16	

Values are expressed in terms of mean ± SEM (*n* = 6)

The bands at 2924 cm^−1^ in both formulations (Fig. 4b, c) correspond to stretching vibrations of CH_3_ and CH_2._ The characteristic peak at 1629 and 1639 cm^−1^ (amide I) in CNPs and PM-CNPs, indicates the formation of nanoparticle due to the binding of proteins with O and N of those groups [49]. The presence of P=O peak can be observed at 1149 cm^−1^ in both CNPs and PM-CNPs, due to the prospective interaction of protonated amine/amide groups of chitosan and negatively charged polyphosphoric groups of sodium polyphosphate, which enables enhancing both inter and intramolecular interactions in CNPs [50]. The characteristic peaks at 1464 and 1377 cm^−1^ ascribes to the aldehyde groups in which hydroxyl end groups of heartwood extract was transformed after the oxidation reaction in PM-CNPs (Fig. 4c) formulation.

A peak at 1529 and 1618 cm^−1^ in *Pterocarpus marsupium* heartwood extract (Fig. 4a), represents the presence of the ketone and carboxylic acid group –C=O, respectively, due to the presence of terpenoids, tannins, and flavonoids. The peak at 2341 cm^−1^ is due to the bending vibration of alkynes. The broad absorbance peak at 3437 cm^−1^ is correlated with the presence of hydroxyl functional groups in the phenolic and alcoholic compounds.

### Shape and surface morphology

The surface morphology of the CNPs and PM-CNPs was determined by SEM (Fig. 5). There was a large amount of *nearly* separated spherical nanoparticles as observed by Yang and Gan Q [51] and Wang [52]. The unloaded chitosan nanoparticles have a particle size of 80–90 nm and the size of PE-loaded chitosan nanoparticles is of 100–110 nm. Hence, the size of the nanoparticle increases by the addition of plant extract. Notably, the hydrodynamic diameter of particles measured by DLS was larger than the size calculated by microscopy generally due to the high capacity of CNPs to swell. In DLS, we obtain the particle hydrodynamic radius while we get an estimate of the observed area diameter by SEM. For DLS, as a distributed particle moves through a liquid medium, the solvent adheres to its surface through a thin layer of an electric dipole. This layer determines the particle movement throughout the medium.

Hydrodynamic diameter, therefore, gives us knowledge of the inorganic center along with the coating substance and the solvent layer bound to the surface as it passes under the influence of Brownian motion. At its core, DLS offers excellent statistics for an average size (by intensity), average poly-dispersity index (PDI), and a moderately peak-resolved statistical inversion distribution. While estimating size by SEM, this layer of hydration was therefore not present, so we only get information about the inorganic core. The variation occurs when DLS tests the diffusion in water, and even the particles of dust in the sample that may alter the readings. So in DLS study, we get a greater size of nanoparticles. TEM pictures also showed that PM-CNPs were spherical as shown in Fig. 6.

### Entrapment efficiency

The entrapment efficiencies of PM-CNPs-1, PM-CNPs-2, and PM-CNPs-3 are 68.87 ± 3.1, 82.78± 5.67, and 86.76± 5.43 as shown in Table 1. Beyond PM-CNPs-3 extract concentration, entrapment efficiency almost remained constant. This can be explained by the fact that initially nanoparticles were not saturated with the drug; the increase in drug concentration increased the availability of the drug for encapsulation. An increase in the concentration of drug encapsulated increased the size of the nanoparticles. Further enhanced concentration of drug shows no further drug encapsulation, keeping the size of the particle constant. The size of the nanoparticle also influences the entrapment efficiency, as larger nanoparticles have a larger volume that can hold more amount of drug. Previous reports have also shown improved EE in ketoconazole-loaded CNPs with an increase in the mean particle size of the nanoparticles [53].

**Table 3:** Inhibition of albumin denaturation by PM-CNPs-2

Treatment	Concentration (μg/ml)	Percentage of inhibition	IC_50_
Chitosan nanoparticles	20	19.23 ± 1.07	92.76
Loaded *Pterocarpus*	40	37.24 ± 1.36	
*Marsupium*	60	49.37 ± 1.27	
	80	56.76 ± 1.65	
	100	63.52 ± 1.42	
Diclofenac sodium	20	12.87 ± 1.02	157.43
	40	37.26 ± 1.47	
	60	56.35 ± 1.52	
	80	73.33 ± 1.68	
	100	89.90 ± 2.01	

Values are expressed in terms of mean ± SEM (*n* = 6)

**Table 4:** Different indicated criterion results of chitosan nanoparticle formulations

Formulation	Plant extract concentration (***μl***)	Zeta potential (Mv)	Particle size (nm)	PDI
CNPs	0	36.2	238.7± 1.45	0.276
PM-CNPs-1	5%	40.4	445± 2.43	0.365
PM-CNPs-2	10%	57.3	676 ± 2.76	0.465
PM-CNPs-3	15%	44.6	547±2.86	0.343

**Table 5:** FT-IR peaks of *Pterocarpus marsupium*-loaded chitosan nanoparticles and their respective assigned functional groups

S. No.	FT-IR peaks in PM-CNPs (cm^−1^)	Functional groups
1.	3402.54	Presence of hydroxyl bond
2.	2924.18	Stretching vibrations of CH_3_ and CH_2_
3.	1639.55	Presence of C=O stretch in an amide bond
4.	1464.02	CH_3_ and CH_2_ deformations
5.	1377.22	Symmetrical bending of CH_3_
6.	1149.01	Presence of P=O peak
7.	1047.36	Stretching vibration of –C–O–

### In vitro drug release studies

For drug release studies, PM-CNPs-2 has been chosen. UV-spectrophotometric assessment evaluated the amount of plant extract released from nanoparticles using the calibration curve. Chitosan nanoparticles imparted sustained release to the drug wherein the drug was released up to greater than 85% from PM-CNPs in 15 h. The release rates of PM-loaded nanoparticles showed an initial burst release [54] of about 50% in the starting 8 h with a subsequent release of 35% in the next 8 h at a slower pace. In the initial phase, the burst release was quickly released into the medium due to drug adsorption on the surface of the chitosan nanoparticles. In the next phase, the slow release was due to the slow degradation of chitosan nanoparticles by diffusing the release medium inside the NPs, allowing the diffusion of entrapped drugs through nanoparticle pores into the medium. The in vitro PM release from PM-CNPs-2 is demonstrated in Fig. 7 from which it was proven that the release of the drug from chitosan nanoparticles was sustained throughout 15 h with a cumulative release of 37.5%.

### Amylase inhibition

Alpha-amylase is an enzyme involved in starch breakdown, and the inhibition of amylase activity is a further therapeutic target in controlling type II diabetes. Several studies centered on pterostilbene and (-)epicatechin which were recognized as two main compounds in PM heartwood and bark responsible for antidiabetic effects. Antidiabetic drugs attack complex biochemical pathways including carbohydrate digestion. α-amylase and α-glucosidase are the main enzymes involved in the digestion of dietary carbohydrates into easily absorbable molecules. Inhibition of these enzymes is beneficial in reducing postprandial blood glucose spikes [55]. ZnO nanoparticles-loaded *Pterocarpus marsupium* extract was analyzed for alpha-amylase activity in a concentration range of 20–100 μg/ml, and the IC_50_ was found to be 59.38 [56].

### Inhibition of protein denaturation

Protein denaturation implies the loss of biological characteristics of protein molecules, their structure, and function. Therefore, any plant material that prevents protein denaturation may be an effective anti-inflammatory agent. Proven studies showed the effectiveness of *P. marsupium* extract towards the anti-inflammatory activity. Methanol and aqueous extracts of *Pterocarpus marsupium* showed similar anti-inflammatory potential determined by their IC_50_ values of 45 ± 1.6 μg/ml, 45 ± 0.94 μg/ml, compared to 55 ± 0.24 μg/ml for diclofenac sodium [57]. *P. marsupium* methanol (50 mg/kg.b.wt) and aqueous extract (100 mg/kg.b.wt) was administered to carrageenan mediated rat paw edema model to test anti-inflammatory activity. Both the extracts were found to have significant anti-inflammatory activity [58, 59]. *P. marsupium* aqueous extract was found to decrease elevated inflammatory cytokine, TNF-α in NIDDM diabetic rats at doses of 100 mg/kg and 200 mg/kg.b.wt [60]. Anti-inflammatory therapy is promising for drugs or phytochemicals that are useful in stopping protein denaturation [61]. Phenolics and flavonoids present in plant extracts mediate the anti-inflammatory activity [62]. In this research, the chitosan nanoparticles noted a dose-dependent inhibition of protein denaturation, exhibiting its anti-inflammatory activity. The ability to inhibit protein denaturation helps to mitigate indirectly, and this can help to develop anti-inflammatory medicines.

## Conclusion

The method used for the synthesis of chitosan nanoparticles using *Pterocarpus marsupium* is a novel, cost-effective, and is ecofriendly for synthesizing CNPs compared to well-known established methods showing high particle yield at low concentration of plant extract. The synthesized CNPs are highly stable. The XRD analysis showed less intense peaks of PM-CNPs due to the cross-linking of chitosan with TPP and plant extract. The FT-IR spectrum proves the formation of chitosan nanoparticles loaded with *Pterocarpus marsupium* extract. The shape and morphology of the synthesized nanoparticles assessed by SEM and TEM which demonstrate its near-spherical nature with size ranging between 100 and 110 nm. The phytosynthesized chitosan nanoparticles have shown enhanced EE for plant extract with a sustained release characteristics of up to 15 h. The synthesized nanoparticles were further researched for a few biomedical applications. The in vitro inhibition of alpha-amylase activity and protein denaturation by the synthesized chitosan nanoparticles makes it an effective therapeutic agent against diabetes and inflammatory disorders in drug delivery applications.

## Data Availability

Not applicable

## Abbreviations

CS: Chitosan
TPP: Sodium tri-polyphosphate
CNPs: Chitosan nanoparticles
PM: *Pterocarpus marsupium*
PE: Plant extract
PM-CNPs: *Pterocarpus marsupium*-loaded chitosan nanoparticles
EE: Entrapment efficiency
DLS: Dynamic light scattering
PDI: Polydispersity index
FT-IR: Fourier transform infrared spectroscopy
SEM: Scanning electron microscopy
TEM: Transmission electron microscopy
DMF: Dimethylformamide
IC_50_: Half-maximal inhibitory concentration
